# Predictors of Functional School Outcome in Children With Pediatric Acquired Brain Injury

**DOI:** 10.3389/fneur.2022.872469

**Published:** 2022-04-14

**Authors:** Jan Stubberud, Ruth Hypher, Anne E. Brandt, Torun G. Finnanger, Eva Skovlund, Stein Andersson, Kari Risnes, Torstein B. Rø

**Affiliations:** ^1^Department of Psychology, University of Oslo, Oslo, Norway; ^2^Department of Research, Lovisenberg Diaconal Hospital, Oslo, Norway; ^3^Department of Clinical Neurosciences for Children, Oslo University Hospital, Oslo, Norway; ^4^Children's Clinic, St. Olavs Hospital, Trondheim University Hospital, Trondheim, Norway; ^5^Department of Clinical and Molecular Medicine, Norwegian University of Science and Technology, Trondheim, Norway; ^6^Department of Public Health and Nursing, Norwegian University of Science and Technology, Trondheim, Norway; ^7^Psychosomatic and CL Psychiatry, Division of Mental Health and Addiction, Oslo University Hospital, Oslo, Norway

**Keywords:** fatigue, school, cognition, pediatric acquired brain injury, disability

## Abstract

**Objective:**

Among the variety of domains that may be impacted after pediatric acquired brain injury (pABI) are functional school outcomes. The purpose of this study was to identify demographic, medical, and psychological factors associated with impairments in functional school outcomes, defined as school absence, need of educational and psychological services, quality of life (QoL) in the school setting, and academic performance in children with pABI, with a specific emphasis on the significance of fatigue.

**Materials and Method:**

We used baseline data from a randomized controlled trial. The sample consisted of seventy-six children aged 10 to 17 (*M* = 13 yrs) with pABI in the chronic phase (>1 year). All completed assessments of school-related QoL, academic performance, global functioning, fatigue, IQ, behavioral problems, and executive function.

**Results:**

Fatigue, IQ, global functioning, behavioral problems, and sex emerged as potential predictors for functional school outcomes. Of note, overall fatigue emerged as the strongest potential predictor for parent-reported QoL in school (β = 0.548; *p* < 0.001) and self-reported QoL in school (β = 0.532; *p* < 0.001).

**Conclusions:**

Following pABI, specific psychological, medical, and demographic factors are associated with functional school outcomes. Neither of the injury-related variables age at insult and time since insult were associated with functional school outcomes. Overall, our findings may suggest that a reintroduction to school with personalized accommodations tailored to the child's specific function and symptoms, such as fatigue, is recommended.

## Introduction

Pediatric acquired brain injury (pABI), such as traumatic brain injury (TBI) or non-traumatic injuries (e.g., brain tumor, stroke, hypoxia, or infections /inflammation to the brain), is one of the leading causes of lifelong disabilities in school age children (1, 2). Consequences may include persistent impairments in cognition, emotional health, adaptive behavior, and school functioning (3–11). Functional school outcome is of particular concern, here broadly defined as school absence, aid from Educational and Psychological Service (EPS), quality of life (QoL) in the school setting, and academic performance in children (12, 13). School is the principal location for the development of not only academic skills such as mastering the school curriculum, but also cognitive, social, and community-related skills during childhood. For many, return to school life after pABI represent an indicator of a return to normality (14). However, despite significant improvements in medical treatment after pABI, functional school impairments often emerge over time, for example when returning to school after cancer treatment, and are characterized by poor school performance, high rates of grade retention, and need of external educational services (10, 11, 15–17).

For many children, pABI can be viewed as a chronic disease process that initiates ongoing and possibly lifelong changes that influence several organ systems, physical and sensory limitations, in addition to neurocognitive impairment, emotional distress, and fatigue, that may have a cumulative negative effect on functional school outcome. Indeed, functional school outcomes may be influenced and explained by multiple variables and factors which display the complex and interdependent relationship between demographic [e.g., age and sex; (18, 19)], medical [e.g., injury-related variables such as age at injury, time since injury and functional outcome; (20)], and psychological variables [e.g., IQ, executive function (EF), behavioral problems, fatigue; (9, 21–24)].

Typically, functional school outcome is negatively affected during the initial 6 months after pABI, when compared to healthy children or children with orthopedic injuries (25, 26). While some aspects may improve in the first 6 to 12 months after injury, longitudinal studies indicate significant long-term impairments several years after pABI [e.g., (11)]. Age at injury is also a known predictor of outcome (27). Early injury [i.e., 7 years or younger; (28)], has been associated with poorer neurocognitive functions including IQ, attention, memory and EF, in addition to persisting disability (22, 23, 29, 30).

Importantly, neurocognitive and behavioral impairments may have adverse effects on school outcomes (9, 24). Post-pABI cognitive sequelae include deficits in IQ, attention, EF, memory and learning, and language skills (31–33). In particular, concurrent and longitudinal associations between EF and functional school outcomes have been demonstrated, with EF also being longitudinally predictive of academic difficulties and school dropout (34). Executive functions can be described as distinct, but related, higher-order neurocognitive processes responsible for purposeful, goal-directed behavior (35, 36). Relatedly, children with pABI are at risk for persistent symptoms of behavior problems, with potential debilitating effects on children's long-term functioning [e.g., (9, 26, 33, 37, 38)].

Demographic, medical, and psychological factors are central factors influencing functional school outcomes for children with pABI. However, fatigue has been largely overlooked as a potential predictor of functional school outcomes, despite being described as one of the most universal and debilitating symptoms following pABI (39–41), that may have adverse effects on academic outcomes. The etiology of fatigue after pABI is complex, primarily relating to changes in the central and peripheral nervous system and endocrine disturbances (42, 43). In addition, fatigue is related to exacerbating factors such as emotional disorders and cognitive impairment, and in particular executive dysfunction (42, 44). Previous research suggest that fatigue is associated with decreased QoL, interfering with everyday activities (e.g., social and physical activities) and school function (41, 45–48). There is preliminary evidence to suggest an association between fatigue and unfavorable functional school outcomes (i.e., schoolwork being negatively affected and worse academic performance) (46, 47, 49). Beyond these studies, the associations between fatigue and functional school outcome are scarcely examined in the pABI population. However, studies of other conditions provide support to the relationship; for example, there is evidence showing that fatigue is associated with worse cognitive and academic outcomes in pediatric multiple sclerosis (50), as well as with unfavorable functional outcomes of young adult cancer survivors and stroke patients (51, 52). Similarly, disadvantageous social outcomes relating to employment and substantial government benefits in long-term survivors of pediatric brain tumors have been found to be strongly associated with fatigue and executive dysfunction (31). Furthermore, Berrin et al. (53) observed that fatigue is an important determinant in understanding how diagnostic subtypes of cerebral palsy translates into problems with school functioning.

There are, however, several methodological shortcomings in existing studies on functional school outcome in the field of pABI, such as small sample size, inferior measures of fatigue (e.g., yes/no questions) and too narrow definition and assessment of functional school outcome (e.g., no inclusion of objective functional school data), limiting the generalizability and validity of findings. Importantly, most studies evaluating functional school outcome in the context of post-pABI fatigue have been limited by the use of relatively narrow definitions of functional school outcome, such as describing it merely in terms of school performance/work (46, 47, 49). Beyond performance/work, domains of functioning including QoL in the school setting and objective functional school data, such as information regarding school absence and aid from external educational services, have not been formally investigated in children with pABI. To our knowledge, functional school outcome in relation to fatigue after pABI has only been assessed with questionnaires (46, 47) and interviews addressing school performance/work (54). Notably, no studies have examined the association between fatigue and different categories of specific functional school outcomes, or examined these associations in children aged 10–17 years, including both TBI and non-traumatic brain injuries.

In sum, there is an urgent need to employ a broader, more holistic approach to assessing functional school outcome and identify potential predictors of functional school outcome in children with pABI that may assist in developing evidence based personalized interventions to advance school functioning. Thus, the purpose of this study was to investigate how demographic, medical, and psychological factors, with a specific emphasis on fatigue, are associated with impairments in functional school outcomes in children with pABI. Given the lack in previous studies, the present study will have a more exploratory approach and attempt to answer the following questions:

1. Do Demographic, Medical, and Psychological Factors Predict Impairments in Functional School Outcomes, Indicated by School Absence, aid From the EPS, Self- and Parent Reported QoL in School Setting, and Academic Performance?
2. Do Parent-Reported Fatigue Emerge as a Significant Predictor for any of the Functional School Outcomes?

## Materials and Methods

This cross-sectional study presents baseline data derived from a dual site, evaluator-blinded, parallel group randomized controlled trial (RCT) on the efficacy of cognitive rehabilitation for children and adolescents with pABI (55, 56). The original RCT was preregistered at clinical.trials.gov (NCT03215342), approved by the Regional Committee for Medical and Health Research Ethics (2017/772), Norway, and conducted in accordance with principles of Good Clinical Practice, the Helsinki Declaration and the standards for Ethical Research Involving Children (ChildWatch International and UNICEF).

### Participants and Design

Seventy-six participants (mean age 13.4 years, *SD* = 2.3, 57% girls), with pABI resulting from non-traumatic (brain tumor, stroke, hypoxia/anoxia, and brain infections/inflammations; *n* = 58) and traumatic (TBI; *n* = 18) injuries, and who were between the age of 10 to 17 years at time of invitation, were recruited from trauma referral centers from the north, mid- and south-east regions of Norway (see Table 1 for demographic and medical characteristics). Information related to the inclusion and exclusion criteria was collected by a semi-structured interview [for more details about the semi-structured interview see (56)]. Inclusion required executive complaints in daily life as determined by a free description of the child‘s function in daily-life and specific EF questions (e.g., “*Does the child handle doing more than one thing at the same time? Does the child manage to plan activities? Is the child easily distracted?*) in the semi-structured interview, in addition to minimum 12 months since injury/illness or completion of cancer therapy. The exclusion criteria consisted of: (i) pABI before 2 years of age; (ii) cognitive, sensory, physical or language impairment affecting the capacity to attend regular school (i.e., follow educational goals of peers and regular teaching) and/or complete the intervention; (iii) preinjury neurological disease, severe psychiatric disorder and/or stimulant medication; (iv) recently detected brain tumor relapse [see also (56)].

**Table 1:** Demographic and medical characteristics in study sample.

	**(*n* = 76)**
Age years (*M*/*SD*)	13.4 (2.3)
Sex (Girls/boys)	43/33
Primary injury, *n* (%)
Brain tumor	29 (38)
Traumatic brain injury	18 (24)
Cerebrovascular accidents	17 (22)
Inflammation	7 (9)
Anoxia	5 (7)
Cerebral imaging, *n* (%)^a^	76 (100)
Confirmatory pathological findings, *n* (%)^b^	67 (88)
Neurological impairments, *n* (%)	33 (43)
Admission to intensive care unit, *n* (%)	49 (64)
Brain surgery in the brain tumor group	25 (86)
Chemotherapy	11 (14)
Radiation therapy	8 (11)

a *All had conducted magnetic resonance imaging (n = 65) or computed tomography (CT; n = 11) at some point*.

b *Out of the 9 individuals without confirmatory imaging (normal), six had traumatic brain injury, One anoxia, and two with brain infection/inflammation. Three out of these nine had conducted a CT only and all had traumatic brain injury*.

Potential participants were identified based on discharge diagnosis and received a written invitation (*n* = 223). In the case of a positive response, written informed consent was obtained from participants (>16 years) or primary caregivers (participants <16 years). Following this, a semi-structured screening interview was conducted to determine eligibility for study inclusion. Parents reported for participants <16 years of age, and participants older than 16 years of age could attend the interview [for more details about the semi-structured interview see (56)]. Ninety-nine individuals were eligible for screening. Out of these, ten participants did not meet inclusion criteria (i.e., 9 with insufficient EF complaints, and 1 was excluded based on information indicating obvious violation of eligibility not previously communicated) and were as such excluded, while two participants declined to participate. Following randomization (*n* = 87), 76 participants completed a baseline assessment. Pre-inclusion attrition comprised 11 participants (e.g., due to worsening of illness) (see also 57). In the present study, the children were assessed pre-intervention (baseline) at the hospital during one workday by experienced test-technicians, a study nurse, and psychology students (master level) under the supervision of clinical neuropsychologists. To compensate for the potential variation associated with several assessors and to ensure consistent results for the study, a Standard Operating Procedure (SOP) described the protocol and procedures for the assessments, and the test administrators received training from experienced clinical neuropsychologists in addition to being blinded to the type of intervention to be received.

### Measures

#### Demographic and Medical Characteristics

Demographic information, in addition to school absence and aid from EPS, was collected in a structured interview. Medical and injury characteristics were extracted from medical records. This data included brain imaging findings [computerized tomography (CT), magnetic resonance imaging (MRI)] from the first year post injury or disease onset, data regarding admission to intensive care unit, and treatment (e.g., chemotherapy, radiation therapy, brain surgery in brain tumor group). Neurological status (cranial nerve, motor function, balance, sensibility), was obtained in a medical examination by a physician.

#### Functional School Outcomes

##### School Absence

School absence was measured by asking parents: “To what degree has your child been absent from school during the last 6 months?” The degree of absence was indicated using the following response set: <10, 10–50 or >50%. Due to a very low number of responses indicating >50%, we ended up using categories <10 and >10% for the present study.

##### Aid From the Educational Psychological Service

Typically, EPS provides assessments and advice regarding special educational needs in the school setting. In the present study, parents were asked the following question in order to obtain data on EPS aid: “Has your child received any support from EPS?” The following two categories were employed: “no aid” and “current or previous aid.”

##### Quality of Life in the School Setting

Quality of life in the school setting was investigated by employing the 5-item School Functioning subscale from the Pediatric Quality of Life Inventory (PedsQL parent report) (57). The format, instructions, Likert response scale, and scoring method are identical to the PedsQL MFS, with higher scores indicating better QoL in the school setting. Items on the PedsQL school scale assess problems regarding “keeping up with schoolwork,” “paying attention in class,” and “forgetting things.” Furthermore, the scale score has good reliability (α = 0.72) and validity in several health conditions, including pABI (58–60). Finally, good internal consistency (α = 0.93) was demonstrated for the PedsQL (parent) in the present study.

##### Academic Performance

The participants' teachers were administered the Teacher's Report Form ages 6-18 (TRF/6-18) from the Achenbach System of Empirically Based Assessment (ASEBA; 62) to assess the children's academic performance. The TRF/6-18 provide scores for the child's current performance in academic subjects. Here, teachers rate the child's school performance using a 5-point scale ranging from 1 (far below grade level) to 5 (far above grade level) for each academic subject. For adaptive characteristics in school, teachers rate the child on 7-point scales (“Much less,” “Somewhat less,” “Slightly less,” “About average,” “Slightly more,” “Somewhat more,” “Much more”) in four areas: dedication to schoolwork, appropriateness of behavior in school, ability to learn, and happiness. Acceptable internal consistency was found for academic performance (α = 0.72).

#### Potential Predictors

##### Fatigue

Symptoms of fatigue were measured using the 18-item Pediatric Quality of Life Inventory-Multidimensional Fatigue Scale (PedsQL MFS, parent report) (60). It is comprised of three dimensions, General, Sleep/rest and Cognitive fatigue, as well as a total score. The total score was used as outcome in the present study. Items are rated on a 5-point scale (“Never,” “Almost Never,” “Sometimes,” “Often,” and “Almost Always”). Respondents are asked how much of a problem each item have been during the past month (e.g., “feel too tired to spend time with friends”). Items are reverse scored and linearly transformed to a 0–100 scale, so that higher scores indicate better QoL (i.e., less fatigue symptoms). The scale scores of this measure have demonstrated strong evidence of reliability and validity across various pediatric health conditions, including pABI (61–63). In the present study good internal consistency was found for the parent-report (α = 0.93).

##### Behavioral Problems

Behavioral problems was assessed with the Child Behavior CheckList for ages 6–18 from ASEBA (CBCL/6-18; 62). It consists of 113 questions, scored on a three-point Likert scale (0 = absent, 1= occurs sometimes, 2 = occurs often). Here, a parent is instructed to report on the child's problems. The form yields various subscales and three composite scores, of which Total problems (*M* = 50, *SD* = 10), the sum of scores of all the problem items, i.e., behavioral problems, was employed in the present study. The questionnaire has robust psychometric properties with adequate internal consistency (α = 0.82) (64).

##### Executive Function

The Behavioral Assessment of the Dysexecutive Syndrome for Children (BADS-C; 66) consists of six subtests; the Playing Cards Test, the Water Test, the Key Search Test, Zoo Map Tests 1 and 2, and the Six Part Test. It was developed to reflect different EFs (e.g., shifting, planning and goal-directed behavior, estimation abilities and inhibition) in everyday life and is used as a global measure of EF in the present study. A total age-scaled score is converted to an overall scaled score, ranging from 49 to 146. The scores can be classified functionally like this: impaired performance (overall scaled score range 49–68); borderline performance (overall scaled score range 70–78); low average performance (overall scaled score range 80–88); average performance (overall scaled score range 90–109); high average performance (overall scaled score range 111–119), and superior performance (overall scaled score range 121–146) (65).

##### Intellectual Ability

Full scale IQ was estimated by using six subscales (i.e., Vocabulary, Similarities, Digit Span, Coding, Block Design and Matrix reasoning) from the Wechsler Intelligence Scale for Children- Fifth Edition (WISC-V) (66). The WISC-V is an individually administered test battery (*M* = 100, *SD* = 15, subscales *M* = 10, *SD* = 3).

##### Global Functioning

The Glasgow Outcome Scale Extended, pediatric version (GOS-E) is designed to provide a functional outcome, assessing global disability and recovery after brain injury, i.e., inside and outside the home, capacity for work/school, participation in social and leisure activities, and family and peer interactions (67). It consists of a scale with 19 items and eight levels: Level 1 = dead, Level 2 = vegetative state, Level 3 = low severe disability, Level 4 = upper severe disability, Level 5 = low moderate disability, Level 6 = upper moderate disability, Level 7 = low good recovery, and Level 8 = upper good recovery. The GOS-E has demonstrated good psychometric properties (67, 68), and has been found to be strongly associated with functional independence (69).

### Statistical Analyses

Frequency distributions, means, medians, standard deviations (*SD*), and range were calculated for demographic characteristics, potential predictor variables and functional school outcomes (dependent variables). School absence, aid from EPS, QoL in school setting (parent- and self-report), and academic performance were the dependent variables in separate multiple linear regression equations with demographic, medical, and psychological factors as potential predictor variables. Bivariate correlations between potential predictor variables (independent variables) and functional school outcome variables (dependent variables) were computed using Spearman's rho. Variables showing significant correlations (*p* < 0.05) with the functional school outcome variables were then entered into regression equations. Multiple linear regression models were employed for the continuous dependent variables and logistic regression was used for the dichotomized categorical dependent variables. Preliminary analyses were conducted to ensure no violation of the assumptions of normality, linearity, and homoscedasticity, including Mahalanobis distances to find multivariate outliers (removed if present in each equation) (70). To avoid multicollinearity, independent variables demonstrating correlations of >0.70 with other independent variables were removed from the equation (71). In addition, variance inflation factor and tolerance statistics were checked in relation to collinearity (70). In deciding the strength of the relationships, Cohen's (72) guidelines were employed: r = 0.10 to 0.29 (small), r = 0.30 to 0.49 (moderate), and r > 0.50 (large). All statistical testing used an alpha value of 0.05 (two-tailed). Data analyses were conducted using IBM-SPSS version 26.

## Results

### Sample Characteristics

Seventy-six participants were included in the present study. Demographic and medical characteristics are summarized in Table 1. Brain tumor was the dominant cause of injury (*n* = 29), followed by TBI (*n* = 18), and other etiologies accounted for 29 (38%). Almost all had confirmatory cerebral imaging findings (88%), 43% had clinical neurological findings and 64% had been admitted to an intensive care unit. For the brain tumor group, almost all had conducted brain surgery, 38% had received chemotherapy, and 28% had received radiation therapy.

Scores on potential predictor variables and functional school outcomes are presented in Table 2. The mean age when injured was 8 years (*SD* = 3.6), ranging from 1 to 15 years of age. Mean time since insult was almost 5 years (*SD* = 2.7). Regarding global functioning, the GOS-E yielded a mean score of 5.7 (*SD* = 1.4) placing the majority of the sample in the category “upper moderate disability.” Concerning the PedsQL MFS, the group mean fell below the clinically important cutoff score (<70) (60). The sample displayed general intellectual ability, executive test performance and perceived behavioral functioning within the normal range, i.e., the group mean was within 1.5 *SD* from the normative scores on the IQ measure (i.e., WISC-V), the executive test (i.e., BADS-C), and the questionnaire CBCL, respectively.

**Table 2:** Scores on potential predictor variables and functional school outcomes.

	**Measure**	***n* (%)**	**Mean (*SD*)**	**Median (range)**
Potential predictor variables				
	Age yrs. (*n* = 76)		13.4 (2.3)	13 (10, 17)
	Sex (girls) (*n* = 76)	43 (57)		
	Age at insult yrs. (*n* = 76)		8 (3.6)	8 (1, 15)
	Time since insult yrs. (*n* = 76)		4.8 (2.7)	5 (1, 12)
	GOS-E (*n* = 73)		5.7 (1.4)	6 (3, 8)
	PedsQL MFS scaled (*n* = 76)		55.3 (19.2)	56.3 (10, 97)
	Est. full scale IQ (*n* = 72)		92.5 (13.3)	92 (67, 122)
	BADS-C overall scaled score (*n* = 75)		83.9 (20.1)	84 (34, 125)
	CBCL (*n* =71; total problems T-score)		57.6 (9.9)	58 (38, 79)
Functional school outcomes				
	School absence <10% (*n* = 76)	48 (63)		
	Aid from EPS (*n* = 75)	46 (60.5)		
	QoL in school setting Parent-report scaled (*n* = 76)		56.1 (22)	55 (15, 100)
	QoL in school setting Self-report scaled (*n* = 75)		58 (20.1)	60 (15, 100)
	Academic performance T-score (*n* = 69)		44 (6.7)	44 (35, 64)

*GOS-E, the Glasgow Outcome Scale Extended; PedsQL MFS, The Pediatric Quality of Life Inventory Multidimensional Fatigue Scale, parent report, total score; BADS-C, Behavioral Assessment of the Dysexecutive Syndrome for Children; CBCL, The Child Behavior Checklist Total problems (M = 50, SD = 10); EPS, Educational Psychological Service; Estimated full scale IQ from Wechsler Intelligence Scale for Children–Fifth edition (M = 100, SD = 15). IQ scores are scaled scores. Higher score represents more daily life difficulties in CBCL. For PedsQL MFS and QoL in school setting higher scores indicate less problems (e.g., fatigue). QoL in school setting, Quality of life in school setting from the 5-item School Functioning subscale from the Pediatric Quality of Life Inventory (parent report). Academic performance scores are from the Teacher's Report Form ages 6-18 (TRF/6-18) from the Achenbach System of Empirically Based Assessment (M = 50, SD = 10). Clinical cutoffs, CBCL and academic performance T ≥ 65; PedsQL MFS and QoL in school setting T <70*.

As seen in Table 2, a majority (*n* = 48) had <10% school absence during the last 6 months, and a majority had received aid from EPS (*n* = 46). The group means for both self- and parent-reports of QoL in school setting were below normal (<70) (60). Finally, academic performance was rated by the participant's teachers to be normal, with a group mean *T*-score of 44 (*SD* = 6.7).

### Bivariate Analyses

Correlations between potential predictors (independent variables) and functional school outcome variables were first examined and are presented in Table 3. Statistically significant correlations are described below, including the strength of the relationships:

**Table 3:** Correlations between functional school outcome variables and potential predictor variables.

	**School absence**	**Aid from EPS**	**QoL school (parent)**	**QoL school (self)**	**Academic performance**
Potential predictor variables
Age	0.182	−0.033	−0.116	−23^*^	0.151
Sex	0.119	−0.187	0.012	−0.226	−0.393^**^
Age at insult	0.204	−0.108	−0.052	−0.185	0.180
Time since insult	−0.138	0.154	−0.069	0.018	−0.103
GOS-E	−0.429^**^	−0.41^**^	0.503^**^	0.352^**^	0.369^**^
PedsQL MFS	−0.454^**^	−0.278^*^	0.742^**^	0.707^**^	0.104
IQ	−0.232^*^	−0.505^**^	0.403^**^	0.177	0.721^**^
BADS-C	−0.114	−0.36^**^	0.273^*^	0.085	0.419^**^
CBCL	0.32^**^	−0.045	−0.463^**^	−0.364^**^	−0.022

*QoL school, Quality of life in school setting from the 5-item School Functioning subscale from the Pediatric Quality of Life Inventory (parent report); PedsQL MFS, The Pediatric Quality of Life Inventory Multidimensional Fatigue Scale parent report, total score; EPS, Educational Psychological Service; GOS-E, the Glasgow Outcome Scale Extended; BDS-C, Behavioral Assessment of the Dysexecutive Syndrome for Children; CBCL, The Child Behavior Checklist Total problems; Estimated full scale IQ from Wechsler Intelligence Scale for Children–Fifth edition. Academic performance scores are from the Teacher's Report Form ages 6–18 (TRF/6-18) from the Achenbach System of Empirically Based Assessment*.

* *Correlation is significant at the 0.05 level (2-tailed)*.

** *Correlation is significant at the 0.01 level (2-tailed)*.

#### School Absence

PedsQL MFS, GOS-E, and IQ were negatively correlated with school absence, indicating that more overall fatigue, lower global functioning, and poorer IQ is associated with less school attendance (*p* < 0.05). CBCL (total problems) was correlated with school absence, indicating that fewer behavioral problems are associated with higher school attendance (*p* < 0.01). All correlations between school absence and potential predictors were moderate except IQ (small).

#### Aid From EPS

GOS-E, PedsQL MFS, IQ, and BADS-C were negatively correlated with aid from EPS, indicating that that lower global functioning, more fatigue, poorer IQ, and worse EF performance is associated with receiving more support from EPS (*p* < 0.05). Moreover, all correlations between aid from EPS and potential predictors were moderate except PedsQL MFS (small).

#### QoL School (Parent)

Higher QoL school (parent) scores were significantly associated with higher GOS-E, PedsQL MFS, IQ, and BADS-C scores (*p* < 0.05), indicating that better parent-reported QoL in the school setting is associated with higher global functioning, lower levels of overall fatigue, higher IQ and better EF performance. Additionally, QoL school scores were negatively associated with CBCL scores, indicating that worse parent-reported QoL in the school setting is associated with more behavioral problems (*p* < 0.01). All correlations between QoL school (parent) and potential predictors were moderate to large except BADS-C (small).

#### QoL School (Self)

Higher QoL school (self) scores were significantly associated with higher GOS-E and PedsQL MFS (*p* < 0.01), indicating that better self-reported QoL in the school setting is associated with higher global functioning and lower levels of overall fatigue. Furthermore, QoL school scores were negatively associated with age and CBCL scores, indicating that poorer self-reported QoL in the school setting is associated with lower age and more behavioral problems (*p* < 0.05). All correlations between QoL school (self) and potential predictors were moderate to large except age (small).

#### Academic Performance

Academic performance was negatively associated with sex, indicating that better academic performance is associated with being female (*p* < 0.01). Moreover, GOS-E, IQ, and BADS-C scores were positively correlated with academic performance, indicating that better academic performance is associated with higher global functioning, higher IQ, and better EF performance (*p* < 0.05). Of note, all correlations between academic performance and potential predictors were moderate to large.

#### *Post-hoc* Analysis

A *post-hoc* correlation analysis was conducted to further explore the relationship between the PedsQL MFS subscales (General fatigue: *M* = 58, *SD* = 22.6; Sleep/rest fatigue: *M* = 59.2, *SD* = 23.7; and Cognitive fatigue: *M* = 49.1. *SD* = 21.6) and functional school outcomes.

All PedsQL MFS subscales were significantly associated with QoL in school (self and parent) and school absence (*p* < 0.05), indicating that lower levels of overall fatigue are associated with better parent- and self-reported QoL in the school setting and lower rates of school absence. General and Cognitive fatigue scores were additionally associated with aid from EPS, indicating that less symptoms of General and Cognitive fatigue are associated with less aid from EPS (*p* < 0.05). All correlations between the PedsQL MFS subscales and potential predictors were moderate to large except between Cognitive fatigue and school absence and Cognitive fatigue and aid from EPS (small).

### Multivariate Analysis

Potential predictor variables showing significant correlations (*p* < 0.05) with the functional school outcome variables were entered as independent variables into regression equations, with the functional school outcome variables as the dependent variables (Tables 4–**6**). When running the model's residual plots as well as normality plots were produced and visually inspected. No violation of the assumptions of normality, linearity, multicollinearity, nor homoscedasticity was detected.

**Table 4:** Logistic regression predicting likelihood of school absence.

				**95% CI for OR**	
	***B***	***p-*value**	**OR**	**Lower**	**Upper**
GOS-E	−0.474	0.077	0.623	0.368	1.053
PedsQL MFS	−0.032	0.087	0.968	0.934	1.005
IQ	−0.003	0.901	0.997	0.944	1.052
CBCL (total)	0.06	0.111	1.062	0.986	1.144

GOS-E, the Glasgow Outcome Scale Extended; PedsQL MFS, The Pediatric Quality of Life Inventory Multidimensional Fatigue Scale parent report; IQ, Estimated full scale IQ from Wechsler Intelligence Scale for Children–Fifth edition; CBCL, The Child Behavior Checklist Total problems. ^*^p <0.05.

#### Potential Predictors of Functional School Outcomes

##### School Absence

The full model containing all predictors was statistically significant, X^2^ (4, *N* = 64) = 17.92, *p* = 0.001. The model explained between 24.4 % (Cox and Snell R square) and 33.7% (Nagelkerke R square) of the variance in school absence and correctly classified 73.4 % of cases. None of the independent variables made a unique statistically significant contribution to the model (Table 4).

##### Aid From EPS

The full model containing all predictors was statistically significant, X^2^ (4, *N* = 68) = 21.11, *p* < 0.001. The model explained between 26.7 % (Cox and Snell R square) and 35.8 % (Nagelkerke R square) of the variance in school absence and correctly classified 75 % of cases. Only IQ made a unique statistically significant contribution to the model [OR.939, 95% CI (0.886, 0.996); Table 5].

**Table 5:** Logistic regression predicting likelihood of aid from EPS.

				**95% CI for OR**	
	***B***	***p-*value**	**OR**	**Lower**	**Upper**
GOS-E	−0.399	0.15	0.671	0.390	1.155
PedsQL MFS	−0.014	0.396	0.986	0.956	1.018
IQ	−0.063	0.035^*^	0.939	0.886	0.996
BADS-C	−0.011	0.557	0.989	0.953	1.026

*GOS-E, the Glasgow Outcome Scale Extended; PedsQL MFS, The Pediatric Quality of Life Inventory Multidimensional Fatigue Scale parent report; IQ, Estimated full scale IQ from Wechsler Intelligence Scale for Children–Fifth edition; BADS-C, Behavioral Assessment of the Dysexecutive Syndrome for Children; CBCL, The Child Behavior Checklist Total problems*.

* *p <0.05*.

##### QoL School (Parent)

A total of 63% of the variance in QoL school (parent) was explained, *F*_(5, 60)_ = 23.1; *p* < 0.001, with PedsQL MFS [β = 0.548; *p* < 0.00; *B* 95% CI (2.131, 4.165)], GOS-E [β = 0.206; *p* < 0.05; *B* 95% CI (1.669, 29.796)], and CBCL total problems [β = −0.205; *p* < 0.05; *B* 95% CI (-1.03, 1.224)] as significant predictors (Table 6).

**Table 6:** Multiple linear regression models with functional school outcome variables as dependent variables and demographic, medical, and psychological factors as independent variables: β, *B*, CIs, *p*-values and adjusted *R*^2^.

**Potential predictor variables**	**QoL school (parent)**					**QoL school (self)**					**Academic performance**				
	**β**	***B***	**95% CI for *B* Lower**	**95% CI for *B* Upper**	***P-*value**	**β**	***B***	**95% CI for *B* Lower**	**95% CI for *B* Upper**	***p***	**β**	***B***	**95% CI for *B* Lower**	**95% CI for *B* Upper**	***p-*value**
Age	-				-	−0.148	−6.55	−14.636	1.526	0.11	-				-
Sex	-				-	-				-	−0.481	−6.442	−8.377	−4.506	<0.001^*^
GOS-E	0.206	15.733	1.669	29.796	0.029^*^	0.151	10.699	−2.471	23.87	0.109	0.060	0.278	−0.506	1.061	0.481
PedsQL MFS	0.548	3.148	2.131	4.165	<0.001^*^	0.532	2.85	1.717	3.983	<0.001^*^	-				-
IQ	0.136	1.124	−0.447	2.696	0.158	-				-	0.663	0.333	0.239	0.427	<0.001^*^
BADS-C	0.015	0.097	−1.03	1.224	0.172	-				-	0.087	0.029	−0.03	0.088	0.33
CBCL (total)	−0.205	−2.273	−4.158	−0.388	0.019^*^	−0.152	−1.569	−3.644	0.505	0.136	-				-
Adjusted *R^2^*	0.63					0.48					0.68				

*QoL school, Quality of life in school setting from the 5-item School Functioning subscale from the Pediatric Quality of Life Inventory (parent report); PedsQL MFS, The Pediatric Quality of Life Inventory Multidimensional Fatigue Scale parent report; EPS, Educational Psychological Service; GOS-E, the Glasgow Outcome Scale Extended; BADS-C, Behavioral Assessment of the Dysexecutive Syndrome for Children; CBCL, The Child Behavior Checklist Total problems; Estimated full scale IQ from Wechsler Intelligence Scale for Children–Fifth edition. Academic performance scores are from the Teacher's Report Form ages 6–18 (TRF/6-18) from the Achenbach System of Empirically Based Assessment*.

* *p <0.05*.

##### QoL School (Self)

For QoL school (self), 48% of the variance was explained, *F*_(4, 63)_ = 16.18; *p* < 0.001, with PedsQL MFS [β = 0.532; *p* < 0.001; *B* 95% CI (1.717, 3.983)] as a significant predictor (Table 6).

##### Academic Performance

A total of 68% of the total variance in academic performance was explained, *F*_(4, 63)_ =34.83; *p* < 0.001, with IQ [β =0.663; *p* < 0.001; *B* 95% CI (0.239, 0.427)] and sex [β = −0.481; *p* < 0.001; *B* 95% CI (-8.377,−4.506)] as significant predictors (Table 6).

## Discussion

The aim of this study was to extend our knowledge about the ability of demographic, medical, and psychological factors, and in particular fatigue, to predict different categories of specific functional school outcomes in a pABI sample. The main finding of this study was that these factors were associated with all functional school variables examined, except school absence. Secondly, fatigue made the strongest unique contribution in explaining self- and parent reported QoL in the school setting.

Return to school after pABI may pose several challenges for families and school personnel. The complex constellation of neurocognitive, emotional and physical symptoms can hamper learning and cause increased absence rates. In the present study, fatigue and IQ emerged as the strongest contributors in explaining less favorable functional school outcomes, followed by global functioning, behavior problems and sex. Indeed, fatigue was the strongest contributor for overall QoL in the school setting (including both parent- and self-reports) when the variance explained by all other variables in the models was controlled for. Specifically, greater fatigue severity was associated with poorer school-related QoL. This is a central aspect of school functioning, and to our knowledge, no previous research has demonstrated these specific associations in a pABI sample with different etiologies. Although more conjectural, the *post-hoc* analysis showing that all dimensions of fatigue (i.e., General, Sleep/rest and Cognitive) were significantly associated with overall QoL in school, suggest that this relationship is driven by several aspects of fatigue. However, a high level of caution is needed in making inferences from the exploratory analyses. In relation to these findings, it is also important to consider the potential overlap in item content between fatigue and QoL measures. The association between fatigue and QoL measures is potentially driving the relationship (i.e., correlations of > 0.7 reported in Table 3). Nevertheless, these findings are in accordance with findings from previous research. Fatigue has been associated with functional school outcomes (i.e., schoolwork being negatively affected and worse school performance) in a variety of pABI types [e.g., (46, 47, 49)]. In addition, post-ABI fatigue has also been associated with health-related QoL, with increased fatigue being associated with greater perceived negative impact of health issues on a range of daily activities in adult ABI populations [e.g., (73)]. In a study by Macartney et al. (49), pediatric brain tumor survivors described how they felt too tired to attend school following pABI, in addition to experiencing cognitive problems (e.g., memory and concentration problems) that contributed to learning difficulties. We were able to extend previous findings by providing preliminary evidence of associations of post-pABI fatigue also being associated with other functional school outcomes (i.e., QoL in the school and school absence), beyond questionnaires and interviews only addressing performance/work employed in previous research [e.g., (46, 47, 49)]. Although none of the independent variables were able to predict school absence, there was a certain trend toward significance for fatigue (*p* = 0.087), and the *post-hoc* analysis showed that all dimensions of fatigue (i.e., General, Sleep/rest and Cognitive) were significantly associated with school absence. In a similar vein, the association between fatigue (PedsQL MFS total score) and aid from EPS may primarily be driven by Cognitive and General fatigue. Although speculative, given the exploratory nature of the analyses, these findings might indicate that fatigue also may be associated with school absence. However, our study included only children with reported EF problems in daily life, which may have contributed to a sample with higher levels of fatigue compared to the general pABI population. Moreover, despite fatigue being one of the most common symptoms across ABI conditions, there is no consensus framework, and importantly no single, valid and reliable instrument for the assessment of fatigue due to its subjective and multidimensional nature (41, 74).

IQ emerged as a potential predictor for aid from EPS and academic performance, suggesting that higher IQ is associated with receiving less aid from EPS and better academic performance. This finding was not unexpected when considering that IQ is vital for independent participation in activities such as education, self-care, and later in life, employment and living independently [e.g., (75)]. Notably, there is broad agreement that there is a moderate to strong association between cognitive or general intellectual ability and educational achievement overall (21, 76, 77). The role of EPS is to ensure that expert assessments are prepared when this is necessary, in order to improve the adaptation of the education for pupils with special needs. Special educational needs typically refer to children with learning problems or disabilities that make it more difficult for them to learn compared to most children at the same age (78). The need for this kind of service is less likely when having a higher IQ (21). A high IQ may indicate a good cognitive reserve (i.e., cognitive enrichment) and may as such may be advantageous after a pABI, with less negative behavioral manifestations (79). Of note, many studies have reported that lower cognitive reserve is associated with worse outcomes after ABI [e.g., (80)]. Our findings are also in accordance with a study by Prasad et al. (20) who found that children with TBI have higher rates of school support services than children with orthopedic injuries and healthy comparison children. In a similar vein, Lahteenmaki et al. (81) reported that children who had been treated for cancer had a greater need for help from extra lessons. It is also important to keep in mind that academic performance may be affected by the adverse effect on the learning opportunities caused by neurological changes due to chemotherapy and radiotherapy, and absence from school due to hospitalization.

Global functioning (GOS-E) emerged as a potential predictor for parent-reported QoL in the school setting. Specifically, worse global function after brain injury was associated with poorer school-related QoL. This finding is perhaps not surprising when considering that the GOS-E was designed to provide a global outcome with developmental specificity in the pediatric population, assessing functional status, independence, and participation in relevant societal roles (67). For example, the physical and/or psychological changes caused by cancer treatment can delay a child's return to school, reduce the desire to attend school, and cause more absence from school (82). It is, however, important to keep in mind that the broad categories in GOS-E may inadequately account for the multidimensional nature of pABI outcomes with limited sensitivity to change within specific functional domains (83). The lack of available normative rates of school absence makes it difficult to interpret our findings and understand the extent and implications. Since school is a fundamental context for development, reduced QoL in this context, with the risk of increased absence, has the potential to produce deviations in normal development (84), adding to the existing risk caused by the injury in a developing brain. Acquired brain injuries can disrupt subsequent brain development, causing significant short- and long-term alterations in several functional abilities (4, 33). Interestingly, only parent-reported, not self-reported, QoL in the school setting was associated with global functioning. This can be a result of parents overestimating the impact of global functioning to QoL in the school setting, or that the children underestimate the impact of global functioning, or a combination of the two. It is, however, important to keep in mind that there are several factors that may contribute to the discrepancy between self- and parent reports (85), such as response bias due to parental anxiety or expectations (86), and reduced awareness (87). The nature of self- and parent reporting is complex (88), emphasizing the need of including healthy controls in addition to self- and parent report in future studies.

Children with pABI, and in particular TBI, are at higher risk than non-injured children of behavioral problems, with potential detrimental effects on children's long-term functioning and QoL [e.g., (9)]. There are many studies that provide data for the CBCL as a valid tool to assess co-occurring emotional and behavioral problems in children (89). In the present study, the overall extent of both emotional and behavioral problems (CBCL total behavioral problems) emerged as a potential predictor for parent-reported QoL in school, suggesting that behavioral problems have adverse effects on QoL in school. Of note, behavioral impairments after pABI is common (37), and may negatively impact school performance and educational progress, by hindering both the continued development of current skills and acquisition of new skills (9, 24, 26, 33, 38, 90). For example, behavioral impairments may put children at risk for ineffective interactions with the environment, leading to poor functional school outcome. In fact, emotional ill health in children has been associated with a range of adverse functional school outcomes such as educational failure and higher rates of school absence (91–93). Although not specifically assessed in the present study, it is important to mention that difficulties in emotional regulation are among the most common consequences of ABI, with potential detrimental effects in all life domains [e.g., (5, 94–96)]. Emotional regulation can be described as an important aspect of EF (97). In our study, EF did not emerge as a significant predictor for any of the functional school outcomes. This is somewhat surprising, as EF include cognitive processes such as shifting, inhibition, and updating of working memory (35, 98), processes that are essential to learning, academic achievement, and behavioral competence [e.g., (34, 99, 100)]. However, there is a lack of consensus on the definition of EF, and the assessment of the construct is a known challenge (e.g., “task impurity”) (101, 102). In particular, the highly structured and examiner-guided setting in which the examination takes place (e.g., BADS-C), makes less demand on the child's goal setting, structuring and decision-making abilities than the real-life setting (101, 102).

Finally, sex emerged as a potential predictor for academic performance (academic subjects and adaptive characteristics in school), suggesting that better academic performance is associated with being female. Previous research has shown that boys perform less well in school assessments when compared to girls, despite similar cognitive test scores (103, 104). There are, however, a couple of issues that needs to be considered when discussing these findings. Although the teachers rate the child's school performance for each academic subject it is possible that there are more complex academic skills not captured by the CBCL (e.g., comprehension, written expression). Moreover, for adaptive characteristics in school, teachers rate the child regarding commitment to schoolwork, appropriateness of behavior in school, ability to learn, and happiness. These areas may be difficult to rate, especially when considering the context of many of these children, being survivors of life-threatening insults, in addition to the consequences following pABI that may include changes in emotional, behavioral, and cognitive functions. Hence, further research into this area is warranted.

Surprisingly, neither of the injury-related variables age at insult and time since insult showed significant correlations with functional school outcome variables. This is not in commensurate with previous research whereby age at insult and time since insult has been associated with functional school outcomes (e.g., 25, 26–28). Most of the cited previous research, however, involved TBI samples, while the present sample consisted of different pABI etiologies with brain tumor as the dominant cause of injury. Additionally, since we included children ranging from 1 to 12 years post-injury, they were at different stages in their recovery processes. Relatedly, the impact of developmental factors has not been studied in the present study. A young brain has the capacity for more efficient neural restitution, by neural regrowth and anatomical reorganization (105, 106), in addition to being more vulnerable to more severe, diffuse and persistent impairments after pABI compared to the adult brain (107, 108). Furthermore, previous research has demonstrated that insult severity (e.g., Glasgow Coma Scale) is as a predictor of outcome after TBI [e.g., (7)]. However, a generally accepted categorization of severity in atraumatic insults is lacking. Since there is no widely recognized measure of injury severity across different etiologies, we were unable to explore the potential impact of insult severity in the present study. Although severe psychiatric disorder was an exclusion criterium and the sample as a whole displayed normal behavioral functioning, some of the participants may have concurrent mental disorders such as anxiety, depression, or posttraumatic stress disorder. Hence, it is recommended to examine factors such as developmental factors, insult severity, and concurrent mental disorders in more detail in future studies.

Research evidence on how to optimize the return to school process after pABI is lacking. Our findings may suggest a reintroduction to school with academic accommodations tailored to the child's specific symptoms, such as fatigue and behavioral problems, and global functioning and intellectual ability (e.g., GOS-E and IQ). Importantly, taking a broader approach to assessing functional school outcomes may be necessary to get a more nuanced understanding of the impact of pABI and to help inform targeted strategies that can support a child's successful return to school and improve educational outcomes following pABI.

### Strengths and Limitations

Strengths of the present study include the large sample size relative to other pABI studies, robust and standardized assessment methods with both child and proxy report and objective functional school data, and the inclusion of both traumatic and non-traumatic brain injuries, increasing the generalizability and validity our findings. However, future research might also want to consider additional fatigue outcomes, such as objective fatigue measures [e.g., (109)]. In addition to the potential overlap in item content between fatigue and QoL measures, factors such as awareness, social desirability bias, response set bias (tendency to respond similarly to all/many items), and over/under reporting of symptoms may influence the accuracy of questionnaire responding. Also, for future research inter-rater reliability should be measured to ensure consistent results. Importantly, our results are based on correlational analyses and self/parent reports, so it cannot be definitively determined whether described predictors had a direct causal impact on the functional school outcomes. In the present study we focused on a restricted number of outcomes. However, there are other variables of interest that may be considered for future research that could impact school outcomes and/or performance on cognitive assessments, such as sleep, pain, parental health and education, other psychosocial and medical variables (e.g., depression, health-related quality of life, social function). Importantly, the present study did not explore the potential impact of injury severity. This should be investigated in future studies. Data regarding level of TBI, type of tumor, cause of cerebrovascular accidents or anoxia should also be collected in future research to better describe the sample. Another important limitation in the present study is that only participants with reported executive difficulties were included, with all being motivated for a cognitive rehabilitation intervention addressing cognitive difficulties. Perceived executive dysfunction may have contributed to a higher fatigue load in the sample. Awareness questionnaires should be considered for future studies. Moreover, a larger sample size may allow for analyses exploring potential between- and within-injury differences. Given the novelty of the study, with very few studies that have examined functional school outcomes and fatigue in a pABI context, a more exploratory approach was employed to the statistical analyses; we did not correct for multiple comparisons. Accordingly, our selection of predictors exceed the recommended predictor/sample size ratio (71). Hence, cautious interpretation of findings is necessitated by the exploratory nature of the analyses and design of the study. Relatedly, regression analyses in a cross-sectional design study with a limited sample also necessitates caution when interpreting findings. Regarding the generalizability of our findings outside of Norway; the participants were recruited from trauma referral centers from the north, mid- and south-east regions of Norway (nationwide), which allows us to generalize the results to other countries with a healthcare and school system comparable to the Norwegian, e.g., the Nordic countries. A potential advantage to our study is that factors such as race, ethnicity, socioeconomic status, health insurance status, care access (rural/urban), and school access/quality inequities do not play a large role in health outcomes in Norway (110). Finally, the cross-sectional design of our study prevents investigation of the trajectory of functional school outcome over time in addition to factors that might influence change in school outcome over time. To expound these matters, future research could include a larger sample and longitudinal methods to examine the course of predictors and school outcomes over time.

### Conclusions

Following pABI, fatigue, IQ, global functioning, behavioral problems, and sex is associated with functional school outcomes. This may suggest that a reintroduction to school with personalized adaptations to the child's specific symptoms, such as fatigue and behavioral problems, and global outcome and intellectual ability is recommended. Importantly, fatigue represents a potentially modifiable treatment target for children with pABI and may, in turn, improve functional school outcome. Finally, out findings support a recommendation to employ a broad approach with information from different sources when assessing functional school outcome to obtain a more comprehensive understanding of pABI.

## Data Availability Statement

The raw data supporting the conclusions of this article will be made available by the authors, without undue reservation.

## Ethics Statement

The studies involving human participants were reviewed and approved by the Regional Committees for Medical and Health Research Ethics, Norway. Written informed consent to participate in this study was provided by the participants' legal guardian/next of kin.

## Author Contributions

JS, RH, AB, TF, ES, SA, KR, and TR have developed study protocol for this study. JS wrote the article with input from all authors. All authors contributed to the final manuscript, including final approval of the version published. Finally, all authors have agreed to be accountable for all aspects of the work.

## Funding

This work was supported by the Norwegian Research Council [Grant Number 260680/H10].

## Conflict of Interest

The authors declare that the research was conducted in the absence of any commercial or financial relationships that could be construed as a potential conflict of interest.

## Publisher's Note

All claims expressed in this article are solely those of the authors and do not necessarily represent those of their affiliated organizations, or those of the publisher, the editors and the reviewers. Any product that may be evaluated in this article, or claim that may be made by its manufacturer, is not guaranteed or endorsed by the publisher.
